# AIM2 Inhibits BRAF-Mutant Colorectal Cancer Growth in a Caspase-1-Dependent Manner

**DOI:** 10.3389/fcell.2021.588278

**Published:** 2021-03-25

**Authors:** Shailendra Shah, Shaolan Qin, Yang Luo, Yizhou Huang, Ran Jing, Jay N. Shah, Jianjun Chen, Huimin Chen, Ming Zhong

**Affiliations:** ^1^Department of Surgery, Patan Hospital, Patan Academy of Health Sciences, Lalitpur, Nepal; ^2^Department of Gastrointestinal Surgery, Renji Hospital, School of Medicine, Shanghai Jiao Tong University, Shanghai, China; ^3^Department of Surgery, Patan Hospital, Patan Academy of Health Sciences, Kathmandu, Nepal; ^4^Division of Gastroenterology and Hepatology, Renji Hospital, School of Medicine, Shanghai Jiao Tong University, Shanghai, China

**Keywords:** colorectal cancer, BRAF-mutant, absent in melanoma 2, caspase-1, patient-derived organoids

## Abstract

Absent in melanoma 2 (AIM2), a DNA sensor that plays an important role in natural immunity system, has been reported to participate in colorectal cancer (CRC) development. However, the functional role of AIM2 in BRAF-mutant CRC remains unclear. In this study, we first investigated AIM2 expression level in BRAF-mutant CRC tumor tissues. Overexpression of AIM2 in CRC cells was performed to investigate the effect of AIM2 on CRC cell viability, and cell death detection and caspase activity assay were performed to explore the mechanism that AIM2 impacts the growth of BRAF-mutant CRC cells. Moreover, we confirmed the antitumor effect of AIM2 in BRAF-mutant CRC cell-derived tumor xenograft (CDX) models as well as patient-derived organoids (PDOs). Herein, we reported that AIM2 expression was lower in BRAF-mutant than that in BRAF wild-type CRC tumor tissues. Restoring the expression of AIM2 in BRAF-mutant CRC cells greatly inhibits the tumor cell growth by inducing necrotic cell death. Mechanism studies revealed that AIM2-induced cell death is in a caspase-1-dependent manner. Additionally, overexpression of AIM2 significantly inhibits tumor growth and metastasis in BRAF-mutant CRC *in vivo*, which was further confirmed in BRAF-mutant CRC PDOs. Taken together, our data suggested that AIM2 inhibits BRAF-mutant colon cancer growth in a caspase-1-dependent manner, which may provide evidence to understand the pathogenesis of CRC with BRAF-mutant, as well as new strategies for manipulation of CRC.

## Introduction

Colorectal cancer (CRC) is one of the most common malignant tumors in humans, resulting in more than 600,000 deaths per year, which accounts for approximately 10% of all cancer-related deaths in the world (Brenner et al., 2014; Bray et al., 2018). BRAF mutation is frequently occurred in CRC, with ∼10% of CRCs harboring a missense mutation at codon 600 (V600E; Cancer Genome Atlas Network, 2012). This mutation results in activating BRAF function and constitutively activates mitogen-activated protein kinase signaling (Wan et al., 2004). Specially, CRC tumors with this type of BRAF-mutant form a distinct molecular subtype with high progression and poor prognosis (Roth et al., 2010; Yaeger et al., 2014). Despite much advancement in diagnosis and treatment for this disease, the prognosis of BRAF-mutant CRC patients is still very poor because of obstinate recurrence and distant metastasis (Eefsen et al., 2015; Malik et al., 2015). Therefore, it is in urgent need to define the molecular mechanisms of carcinogenesis and identify new therapeutic targets for manipulation of BRAF-mutant CRC.

Absent in melanoma 2 (AIM2) is a member of the interferon-inducible PYHIN (PYRIN and HIN200 domain-containing) family (Asefa et al., 2004; Choubey et al., 2010; Schattgen and Fitzgerald, 2011). Functionally, it participates in the innate immune to protect human body by defensing exogenous and endogenous pathogens (Lugrin and Martinon, 2018). It mainly resides in cytoplasm and acts as a DNA senor to bind to double-stranded DNA and recruit the adapter molecule ASC (apoptosis-associated speck-like protein containing a caspase recruitment domain) (Hornung et al., 2009). The recent finding suggested that AIM2 interacting with ASC could induce the formation a multiprotein complex called the inflammasome (Man and Kanneganti, 2015). Moreover, the AIM2-induced inflammasome can activate the function of caspase-1, subsequently promotes the maturation of pro-IL-1β and pro-IL-18 into their bioactive forms, and causes a new type of inflammatory cell death called pyroptosis (Schroder et al., 2009; Rathinam et al., 2010).

The function of AIM2 in innate immunity and inflammation has been well investigated, but its role in cancer remains to be elucidated. Interestingly, AIM2 was reported to play a dual role in tumorigenesis (Choubey, 2016). AIM2 was originally considered as a tumor suppressor gene that is frequently repressing in a variety types of tumors, including renal carcinoma, prostate cancer, and hereditary nonpolyposis colorectal cancer (HNPCC)-associated small bowel cancer (Schulmann et al., 2005; Ponomareva et al., 2013; Chai et al., 2018). However, an increasing number of studies indicated that AIM2 may function as an oncogene in non-small cell lung cancer, cervical cancer, Epstein-Barr virus-induced nasopharyngeal carcinoma, and oral squamous cell carcinoma (Choubey, 2016). Recent studies showed that reduced expression and frequent frame shift microsatellite instability of the *AIM2* had been identified in CRC, and the reduced expression of AIM2 was associated with a poor prognosis of CRC patients (Dihlmann et al., 2014; Zhang et al., 2017; He et al., 2020). These findings suggested that AIM2 may act as a suppressor in the development of CRC. However, none of these studies explore the functional role of AIM2 in BRAF-mutant CRC, and further researches are desperately needed to investigate the relationship between AIM2 and BRAF-mutant CRC.

This study reported for the first time the significantly reduced expression of AIM2 in CRC tissue specimens with BRAF-mutant. Results of *in vitro* experiments showed that AIM2 could induce BRAF-mutant CRC cell death, which was associated with the inflammasome factor caspase-1. Furthermore, it has been shown that overexpression of AIM2 could inhibit tumor growth of CRC with BRAF-mutant *in vivo*. What is important is that the results obtained from transplantation of human CRC patient-derived organoids (PDOs) into nude mice demonstrated that restoration of AIM2 expression significantly inhibited the PDOs growth *in vivo*. Our data demonstrate an important role of AIM2 in the development of CRC with BRAF-mutant and pave a new avenue to treat CRC with BRAF-mutant.

## Materials and Methods

### Patients and Samples

Tumor tissues of CRC patients were obtained from surgical resection in Renji Hospital Affiliated School of Medicine, Shanghai Jiao Tong University. The fresh tissue samples were collected in liquid nitrogen and then prepared in paraffin sections. Tissue samples were obtained with informed consent from patients. All patients were pathological diagnosed with colorectal carcinoma and did not receive chemotherapy or radiation before operation. The study was approved by the Research Ethics Committee of Renji Hospital, and all participants were in agreement with the institutional guidelines. In total, 50 patients were included (25 CRC with BRAF mutation and 25 CRC without BRAF mutation).

### Immunohistochemistry

Standard streptavidin-peroxidase method was performed for immunohistochemistry. Briefly, tissue samples were fixed, embedded, deparaffinized, and rehydrated, and peroxidase block solution was used for 10 min. Then, anti-AIM2 primary antibody was incubated with slides at 4°C overnight. After washing, the sections were incubated with secondary antibodies for 30 min at room temperature. Then, horseradish peroxidase-labeled streptavidin working fluid was used. We used phosphate-buffered saline (PBS) to replace the primary antibody for the negative control. Results were recorded with Leica DM6000 B (Leica Microsystems, Wetzlar, Germany). Scoring was conducted based on the percentage of positive-staining cells: 0–5% scored 0, 6–35% scored 1, 36–70% scored 2, and more than 70% scored 3; and staining intensity: no staining scored 0, weakly staining scored 1, moderately staining scored 2 and strongly staining scored 3. The final score was calculated using the percentage score × staining intensity score as follows: “-” for a score of 0–1, “+” for a score of 2–3, “++” for a score of 4–6, and “+++” for a score of >6. Low expression was defined as a total score <4, and high expression with a total score ≥4, which had been supplemented in the revised method. And, three independent pathologists analyzed the immunohistochemistry results.

### Cell Culture

The human CRC cell lines HCT29, HCT116, COLO205, and SW480 were obtained from ATCC (American Type Culture Collection). All CRC cell lines were culture in RPMI 1640 with glutamine (Life, CA, United States) supplemented with 10% (vol/vol) fetal bovine serum (Life, CA, United States) and Pen/Strep Amphotericin B (Life, CA, United States) 1%. All cell lines were maintained in an atmosphere of 95% air and 5% CO2 at 37°C.

### Construction of Plasmid Vector and Transfection

To restore the expression of AIM2 in CRC cells, human full-length AIM2 cDNA was inserted into the plasmid vector according to the protocol. Casepase-1 shRNA was designed and constructed to knockdown casepase-1 expression in BRAF-mutant CRC cells. Lipofectamine 2000 (Invitrogen) was used to perform transfection. After 48 h, the transfection efficiency was detected by Western blot analysis.

### Cell Viability Assay

Cells were seeded in 24-well dishes at the density of 50,000 cells/mL the day before transfection by plasmid of overexpressed AIM2. Three days later, cells were washed two times with PBS and fixed with 100% ethanol for 30 min before crystal violet staining. Crystal violet was then resuspended in 33% acetic acid, and OD was read at 590 nm. All experiments were repeated three times.

### Cell Death Detection

Cells were seeded in 24-well dishes as described above. After treatment, cells were labeled with annexin V/7-amino-actinomycin D (7-AAD). Annexin V binding and 7-AAD incorporation were detected by an LSRII flow cytometer (BD Biosciences) according to the manufacturer’s protocol. Data were analyzed by using FlowJo software (Tristar, Ashland, OR, United States).

### Caspase Activity Assay

To assess caspase-1 or caspase-3/7 activity, the FAM-YVAD-FMK or SR-DEVD-FMK fluorescent probes (AbdSerotec, Colmar, France) that can bind to cleaved caspases were used according to the manufacturer’s instructions. Briefly, 250,000 cells were concomitantly incubated with probes for 1 h and then washed two times with PBS. Finally, cells were suspended in apoptosis buffer before flow cytometry analysis.

### Western Blot

Whole-cell lysates were prepared as described previously. Protein lysates were obtained from cells with the whole-cell lysates according to the manufacturer’s instruction. After detecting the protein concentration, the protein mix was separated by sodium dodecyl sulfate–polyacrylamide gel electrophoresis and transferred into a nitrocellulose membrane. After incubation for 2 h at room temperature by 5% non-fat milk in Tris-buffered saline with 0.1% Tween-20, membranes were incubated overnight with primary antibodies. Then, membranes were washed three times and incubated with the secondary antibody for 30 min at room temperature and washed three times before analysis with a chemiluminescence detection kit (Amersham). The following mouse monoclonal antibodys (mAbs) were used: anti-β-actin (A1978) and anti-Flag M2 (F3165) from Sigma-Aldrich, anti-pannexin-1 (H00024145-M07) from Abnova (Tebu, Le Perray en Yvelines, France), anti-LXRb (PP-K8917-00) from Perseus Proteomics, anti-NLRP3 (AG-20B-0014), and anti-caspase-1 (AG-20B-0048) from Adipogen (COGER SAS, Paris, France). We also used rabbit pAbs anti-ASC (AL177) from Enzo Life Sciences, anti-cleaved caspase-3 (9661), anti-cleaved caspase-7 (9491), anti-cleaved caspase-8 (9496), and anti-cleaved caspase-9 (9501) from Cell Signaling Technology (St. Quentin, France). Secondary Abs horseradish peroxidase-conjugated polyclonal goat anti-mouse and swine anti-rabbit immunoglobulins (Jackson ImmunoResearch, Interchim) were also used.

### Quantitative Polymerase Chain Reaction Analysis

Total RNA was extracted from CRC tissues and cells using Trizol (Invitrogen). Using M-MLV reverse transcriptase, random primers, and RNAseOUT inhibitor (Invitrogen), 100–300 ng of RNA was reverse-transcribed into cDNA. Real-time polymerase chain reaction (PCR) was performed to quantify cDNA using Power SYBR Green Real-time PCR kit (Life Technologies) on a Fast7500 detection system (Applied Biosystems, Life Technologies). The relative mRNA levels were determined by using the ΔΔCt method. The PCR results were normalized to cyclophilin A, GAPDH, and β-actin levels.

### *In vivo* Experiments

Athymic male nu/nu mice aged from 6 to 8 weeks were used in this study. Subcutaneous implant model was established by subcutaneous injection at a total cell number of 1 × 10^6^ for either control or stably AIM2-expressed HT29 cells in the right back flank of mice. Tumor diameters were monitored with calipers every 5 days. Metastasis model was conducted by tail vein injection at a total cell number of 1 × 10^6^ for either control or stably AIM2 expressed HT29 cells.

### Patient-Derived Organoid

The patient-derived tumor disassociated and diluted into 250 cells per 25 μL of growth factor reduced Matrigel seeded into plate, and N2 medium containing 10% R-spondin-1, 100 μg/mL noggin (Peprotech, NJ, United States), 1.25 mM N-acetyl cysteine (Sigma-Aldrich), 10 mM nicotinamide (Sigma-Aldrich), 50 ng/mL EGF (Peprotech) and bFGF (Peprotech), 10 nM gastrin (Sigma-Aldrich), 500 nM A83-01 (Sigma-Aldrich), and 3 μM SB202190 (Sigma-Aldrich) were added. The growth medium was refreshed every 2 days, and the cells were passaged by mechanical disruption every 10–14 days with a 1:5 split ratio.

### Statistical Analysis

All statistical analyses were performed by GraphPad Prism 6.0. Data are presented as mean ± SD. Student’s *t*-test, 2-tailed, was used to analyze difference between two groups. In all cases, *p* < 0.05 was considered statistically significant.

## Results

### Decreased AIM2 Expression in BRAF-Mutant CRC Tissues

Increasing evidence indicates that AIM2 plays an important role in CRC, but the functional role of AIM2 in BRAF-mutant CRC remains unclear (Dihlmann et al., 2014; Zhang et al., 2017; He et al., 2020). To investigate the relationship between AIM2 and BRAF-mutant CRC, we first explored the expression level of AIM2 in CRC with/without BRAF mutation by utilizing immunohistochemical analysis. CRC tumor tissues were divided into 2 groups: 25 CRC patients with BRAF-mutant and 25 CRC patients without BRAF mutation, and the clinicopathological features of the patients are described in Table 1.

**TABLE 1:** Clinicopathological characteristics of 50 CRC patients.

Group	*N*
**Gender**	
Male	23
Female	27
**Age (years)**	
<50	16
>50	34
**Lymph node metastasis**	
No	38
Yes	12
**Distant metastasis**	
No	48
Yes	2
**Stage**	
I	8
II	30
III	10
IV	2
**Location**	
Colon	46
Rectum	4

**FIGURE 1:**
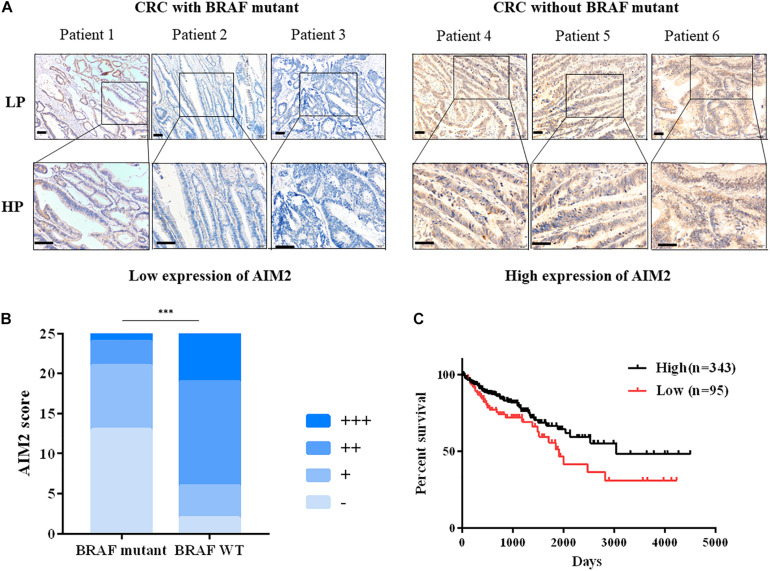
Decreased AIM2 expression in BRAF-mutant CRC tissues. **(A)** Representative immunohistochemical staining of AIM2 in CRC tumor tissues with or without BRAF mutation. Scale bar: 100 μm. **(B)** The quantitative scoring result of AIM2 IHC staining (χ^2^ test, *p* = 0.0002). **(C)** The correlation between AIM2 and CRC prognosis in TCGA cohort [log-rank (Mantel-Cox) test, *p* = 0.02]. ^∗^*p* < 0.05, ^∗∗^*p* < 0.01, ^∗∗∗^*p* < 0.001.

As shown in the Figure 1A, AIM2 was primarily detected in the cytoplasm of intestinal epithelial cell. Moreover, the expression of AIM2 was significantly weaker in CRC tissues with BRAF mutation than that in CRC tissues without BRAF mutation (Figure 1B). Next, we detected the AIM2 expression in BRAF inhibitor PLX8394-treated BRAF-mutant CRC cells, we found that BRAF inhibitor administration enhanced AIM2 expression, indicating that BRAF inhibition could restore AIM2 expression in CRC cell lines (Supplementary Figure 1). Furthermore, TCGA data analysis showed low AIM2 expression in CRC patients associated with poor prognosis (Figure 1C). Our data indicated that AIM2 expression was low in CRC tissues with BRAF mutation, which is associated with poor prognosis.

### Restoration of AIM2 Expression Inhibits the Growth of BRAF-Mutant CRC Cells *in vitro*

**FIGURE 2:**
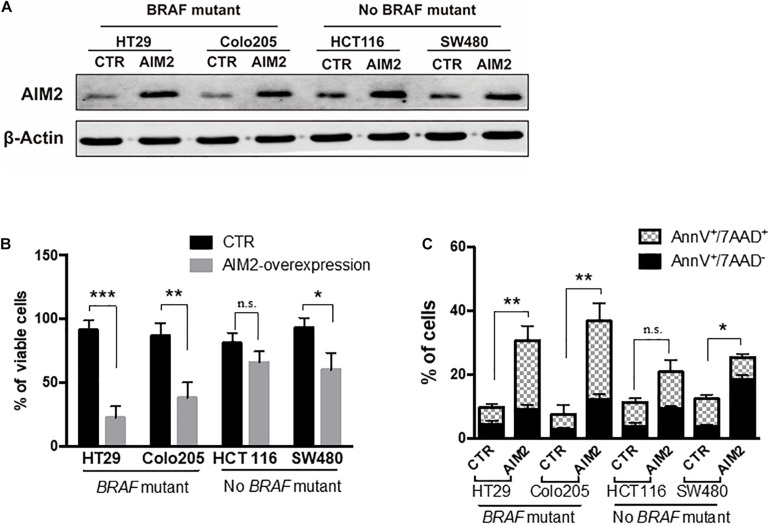
Restoration of AIM2 expression inhibits the growth of BRAF-mutant CRC cells. **(A)** The efficiency of plasmids transfection in human CRC cell lines (HT29, Colo205, HCT116, and SW480) was determined by Western blotting 2 days after transfection. **(B)** Cell viability assay was performed to evaluate the proliferation of human CRC cells after transfection of overexpressed AIM2 plasmid or control vector (*n* = 3, Student’s *t*-test). **(C)** Annexin V/7-AAD staining and flow cytometry analysis were performed to investigate the type of cell death in human CRC cells after transfection of overexpressed AIM2 plasmid (*n* = 3, χ^2^ test *t*). *p < 0.05, **p < 0.01, ***p < 0.001.

We next investigated the functional role of AIM2 in BRAF-mutant CRC cells. We initially tested the effect of restoring AIM2 expression on cell viability in human CRC cells using a plasmid vector. We used four CRC cell lines: two cell lines with BRAF-mutant (HT29, colo205) and two human CRC cell lines without BRAF mutation (HCT116, SW480). Figure 2A shows that the restored AIM2 expression in CRC cell lines was confirmed by Western blot analysis. Cell viability assay was performed 3 days after transfection, and results demonstrated that restoration of AIM2 expression dramatically inhibited cancer cell proliferation in HT-29 and colo205 compared with those in HCT116 and SW480 (Figure 2B). Previous study showed that AIM2 inhibits cell proliferation mainly through inducing cell death (Fernandes-Alnemri et al., 2009). To our knowledge, cell death could be generally divided into two types: apoptosis (also known as programmed cell death that cells with annexin V^+^) and necrosis (uncontrolled cell death that cells with annexin V^+^/7-ADD^+^) (D’Arcy, 2019). As shown in Figure 2C, restoration of AIM2 expression-induced apoptotic cell death was measured by phosphatidylserine (PS) exposure (annexin V^+^cells), and necrotic cell death was measured by cell membrane rupture (annexin V^+^/7-ADD^+^). Our results showed that restored AIM2 expression mainly induced annexin V^+^/7-ADD^+^ cell death in CRC cells with BRAF-mutant (HT29 and Colo25), suggesting that restoration of AIM2 expression inhibited the cell growth by inducing necrotic cell death in BRAF-mutant CRC cells.

### Restoration of AIM2 Expression Induces BRAF-Mutant CRC Cell Death in a Caspase-1-Dependent Manner

**FIGURE 3:**
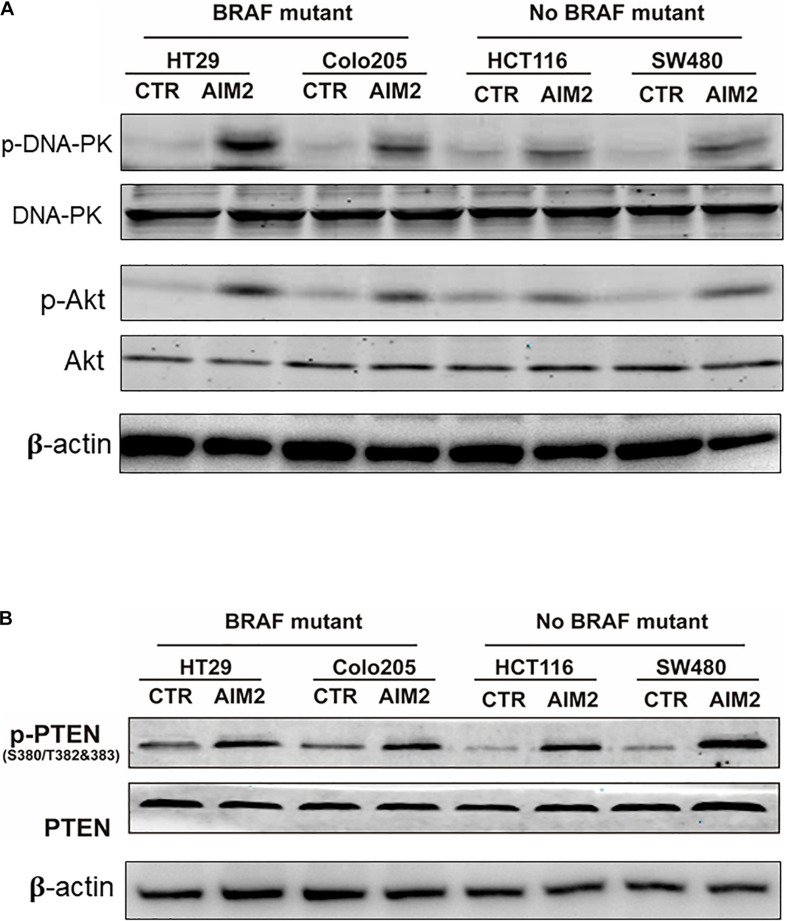
AIM2-induced cell death is not dependent on DNA-Pk/Akt or PTEN pathway. **(A)** Western blot analysis of phosphorylated DNA-PK (p-DNA-PK) and p-Akt in BRAF-mutant CRC cells (HT29 and Colo205) and no BRAF-mutant cells (HCT116 and SW480) after transfection of AIM2 plasmid for 12 h. β-Actin was used as the loading control. **(B)** Western blot analysis of p-PTEN in BRAF-mutant cells (HT29 and Colo205) and no BRAF-mutant cells (HCT116 and SW480) at 12 h after transfection of AIM2 plasmid.

Previous studies reported that AIM2 could inhibit DNA-PK/Akt pathway and promote the activity of PTEN activity to inhibit the activation of Akt and the expression of its downstream genes (Man et al., 2015; Wilson et al., 2015). Thus, we next wanted to figure out whether DNA-PK/Akt pathway or PTEN takes part in the AIM2-induced necrotic cell death in BRAF-mutant CRC cells. After 12 h of transfection with AIM2 plasmid, cell protein lysis was collected to investigate the inhibition of DNA-PK, Akt, and PTEN by Western blot (Figure 3A). The results showed that the alterations of these signaling pathway in BRAF-mutant CRC cells were consistent with those in BRAF wild-type CRC cells. Considering that AIM2 only exhibit effects in BRAF-mutant CRC cells, these data suggested that the DNA-PK/Akt pathway or PTEN was not involved in the AIM2-induced necrotic-like cell death in BRAF-mutant CRC (Figure 3B).

**FIGURE 4:**
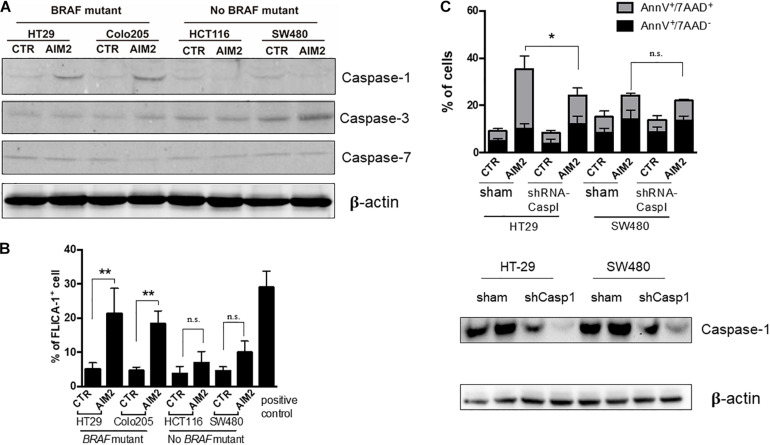
Restoration of AIM2 expression induces BRAF-mutant CRC cell death in a caspase-1-dependent manner. **(A)** Western blot analysis of cleaved caspase-1, -3, and -7 in BRAF-mutant cells (HT29 and Colo205) and no BRAF-mutant cells (HCT116 and SW480) at 12 h after transfection of AIM2 plasmid. β-Actin was used as the loading control. **(B)** Measurement of caspase-1 activation in CRC cells transfected with AIM2 for 12 h by FLICA-1 staining. ATP (5 mM, 1 h) was used as positive controls (*n* = 3, Student’s *t*-test). **(C)** Cell viability was analyzed by annexin V/7-AAD staining in HT29 and SW480 cells transfected with AIM2 for 3 days (*n* = 3, χ^2^ test *t*). Caspase-1 knockdown was confirmed by western blot (WB). *p < 0.05, **p < 0.01, ***p < 0.001.

Besides, caspases have also been reported to participate in AIM2-mediated cell death in cancer cells (Fernandes-Alnemri et al., 2009). To further explore the mechanism of the AIM2-induced necrotic-like cell death in BRAF-mutant CRC, we investigated whether caspases were activated in our model. The result of Western blot, using specific antibodies targeting caspases-1, -2, and -3, showed that restoration of AIM2 expression could activate the caspase-1, whereas no activation of caspase-3 or caspase-7 was detected (Figure 4A). Moreover, this finding was further confirmed by the FILCA fluorescent probes in AIM2 restored BRAF-mutant CRC cells (Figure 4B). In addition, silencing of caspase-1 by shRNA inhibited AIM2-induced necrotic cell death in HT29, showing the more significant effect of caspase-1 silencing on membrane permeabilization (annexin V^+^/7-ADD^+^cells) than on PS exposure (annexin V^+^/7-ADD^–^ cells) (Figure 4C). In summary, these results showed that the AIM2-induced necrotic-like cell death in BRAF-mutant CRC is in a caspase-1-dependent manner.

### Restoration of AIM2 Expression Inhibits BRAF-Mutant CRC Cell Growth and Metastasis *in vivo*

**FIGURE 5:**
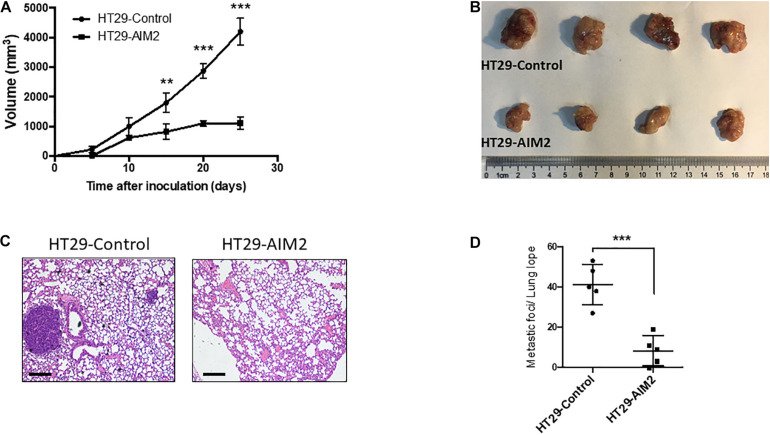
Restoration of AIM2 expression inhibits BRAF-mutant CRC tumor growth and metastasis *in vivo*. **(A)** Tumor growth curves of AIM2 overexpressed HT29 cells and control cells in nude mice (*n* = 4, Student’s *t*-test). **(B)** The picture of tumors isolated from nude mice 25 days after tumor cell injection (*n* = 4). **(C)** Lung metastasis was determined by hematoxylin-eosin staining (*n* = 5). Scale bar: 100 μm. **(D)** The number of lung metastasis foci in nude mice injected with overexpressed AIM2 HT29 cells and control cells (*n* = 5, Student’s *t*-test). *p < 0.05, **p < 0.01, ***p < 0.001.

To further determine whether restoration of AIM2 expression has an inhibition effect on the tumor growth in BRAF-mutant CRC cells, xenograft tumor models were constructed in nude mice with stable expression of AIM2 in HT29 cells. Tumor volumes were measured every 5 days to draw the tumor growth curve, and results showed that restoration of AIM2 expression significantly inhibited the tumor growth of HT29 cells *in vivo* (Figures 5A,B). Furthermore, we also found that restoration of AIM2 expression significantly inhibited tumor pulmonary metastasis, showing the reduced tumor burden in the lung and the number of metastatic foci (Figures 5C,D). Overall, our data demonstrated the restoration of AIM2 expression inhibits the tumor growth and metastasis of BRAF- mutant CRC *in vivo*.

### Restoration of AIM2 Expression in BRAF-Mutant CRC Patient-Derived Organoid Inhibits Tumor Growth *in vivo*

**FIGURE 6:**
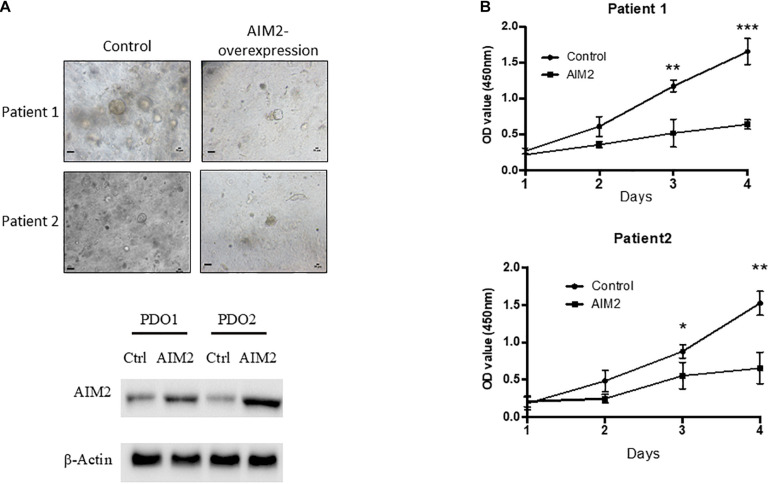
Restoration of AIM2 expression inhibits PDOs growth in BRAF-mutant CRC. **(A)** Two BRAF-mutant CRC PDOs were constructed and transfected with AIM2 overexpression vectors and planted into Matrigel under the HISC condition (WENRg + nicotinamide + A83-01 + SB202190). The morphology of PDOs was recorded 14 days after cell plantation into Matrigel. Scale bar: 100 μm. The AIM2 restoration was confirmed by WB. **(B)** The growth curves of BRAF-mutant CRC PDOs (*n* = 3, Student’s *t*-test). *p < 0.05, **p < 0.01, ***p < 0.001.

Our results demonstrated that AIM2 could inhibit the proliferation and metastasis of BRAF-mutant CRC cells. To further verify the effect of AIM2 on the growth of BRAF-mutation CRC cells, two BRAF-mutant CRC PDOs were constructed and tested. CRC PDO was established as follows: single tumor cell suspension was obtained after digestion of primary tumor tissues and then planted into Matrigel for culture. In this study, we transfected AIM2-overexpression vector into single tumor cells and cultured these cells in Matrigel for 14 days to build the PDOs. Figure 6A shows that restoration of AIM2 expression dramatically inhibited the growth of PDOs in both of these two patients with BRAF mutation. Additionally, we passaged the PDO with AIM2 overexpression and observed the growth curve of these PDOs, and the results also demonstrated AIM2 restoration could inhibit the growth of BRAF-mutant CRC PDOs (Figure 6B), suggesting that restoration of AIM2 expression inhibits the tumor cell growth in CRC patients with BRAF mutation.

## Discussion

Absent in melanoma 2 is a member of the pattern recognition receptor family that is recognized as the key regulator in a variety of diseases, especially cancers (Choubey, 2016; D’Arcy, 2019). Studies also have identified AIM2 as a tumor suppressor in the development CRC, implicating a significant role of investigating the treatment of CRC (Dihlmann et al., 2014; He et al., 2020). Mutation in the BRAF gene at codon 600 (V600E) is an oncogenic event that is reported in approximately 10% of CRC patients (Tejpar et al., 2010). CRC patients are often right-sided and at an advanced stage of disease and a dismal prognosis. Furthermore, these patients are facing limited treatment options, because of its resistance to standard therapies. As AIM2 is reported as a tumor suppressor in CRC, we want to investigate its functional role in BRAF-mutant CRC and try to pave the way for CRC treatment. In this study, we found that the expression level of AIM2 was significantly lower in CRC with BRAF mutation compared with that of CRC without BRAF mutation. In addition, we demonstrated that restoration of AIM2 expression could significantly inhibit BRAF-mutation CRC growth *in vitro*, but not CRC cells without BRAF mutation. Mechanistically, we found that AIM2 induces BRAF-mutant CRC cell death in a caspase-1-dependent manner. Importantly, the growth inhibition mediated by AIM2 on BRAF-mutant CRC was verified in xenograft nude mice model and CRC PDOs. Thus, our study provides a novel insight into the role of AIM2 in BRAF-mutant CRC cell growth and a promising option for CRC treatment. Additionally, Woerner et al. (2007) reported that loss of AIM2 expression is frequent in microsatellite instability CRCs and BRAF mutation always associated with MSI, CIMP, and poor differentiation specimens, suggesting that loss of AIM2 expression in BRAF-mutant CRC may be a result of microsatellite instability.

Recent studies have shown that AIM2 plays an important dual role in innate immunity and tumorigenesis, but its role in cancer is not fully clarified. In consistence with our study, AIM2 was demonstrated to inhibit proliferation and tumorigenicity in breast cancer, renal carcinoma, and prostate cancer (Chen et al., 2006; Ponomareva et al., 2013; Chai et al., 2018). However, AIM2 was also reported to act as oncogene in the development of several cancers, suggesting it is necessary to investigate the role of AIM2 in different cancers (Choubey, 2016). Studies have shown that a reduced expression of AIM2 was found in the CRC tissues and was closely related to a poorer prognosis of CRC patients. Therefore, AIM2 was proposed to be a suppressor in CRC (Wilson et al., 2015). However, the role of AIM2 in BRAF-mutant CRC has not been well investigated. In this study, we reported for the first time the role of AIM2 in BRAF-mutant CRC cells. Our data demonstrated that overexpression of AIM2 could induce necrotic cell death in BRAF-mutant CRC cells by activating caspase-1 function. The silencing of caspase-1 inhibited AIM2-induced cell death in HT29. Activation of caspase-1 and membrane permeabilization increase are the two main features of pyroptosis-mediated cell death. Activation of caspase-1 not only can lead to inflammation, but also can cause an inflammatory form of cell death called pyroptosis, which was characterized by early cell membrane permeabilization and the release of the cytoplasmic content, acting as a signal for neighboring immune cells into the extracellular environment (Wang, 2020). We found AIM2 could modulate the function of caspase-1 in BRAF-mutant CRC cells, suggesting AIM2 inhibits BRAF-mutant CRC cell growth via inducing pyroptosis.

It has been reported that inhibition of DNA-PK/Akt pathway and promotion of PTEN activity could inhibit the activation of Akt and the expression of its downstream genes correlated with proliferation (Wilson et al., 2015). While our study showed for the first time that the BRAF-mutant CRC cell death induced by AIM2 was not dependent on DNA-PK/Akt or PTEN but caspase-1, previous studies demonstrated that caspase-1 could promote cell pyroptosis in the development of colitis and colitis-associated cancer and had importance within colon cancer cells in the effects of liver X receptor agonist on tumoral growth *in vivo* and on cell death *in vitro* (Bergsbaken et al., 2009; Dupaul-Chicoine et al., 2010; Derangere et al., 2014). Besides, the result of *in vivo* experiment demonstrated that the restoration of AIM2 expression could inhibit the tumor progression of BRAF-mutant CRC, including tumor growth and distant metastasis. Furthermore, we also established the BRAF-mutant PDOs and demonstrated that overexpressed AIM2 could significantly inhibit the development of CRC with BRAF mutation, suggesting that pharmacological activation of AIM2 may be beneficial in BRAF-mutant CRC patients.

Although the significant role of AIM2 in BRAF-mutant CRC cells was revealed, several limitations still exist in our study. On the one hand, the conclusion we made that AIM2 expression is low in BRAF-mutant CRC may not be credible as there were only 50 patients analyzed. Thus, more researches with more tissue samples from multicenter will be needed for further confirmation. On the other hand, we found that AIM2 inhibits BRAF-mutant CRC cell growth mediated by caspase-1 activation to speculate that pyroptosis is involved in this process, and more efforts need to be exerted to verify this finding in future studies. In addition, this study merely explored the relationship between AIM2 and BRAF-mutant CRC, and the specific mechanisms of the activation of caspase-1 or its downstream molecule still need to be further researched.

In summary, our results demonstrated that AIM2 expression was low in BRAF-mutant CRC, and restoration AIM2 expression in BRAF-mutant CRC cells could inhibit cancer cell growth in a caspase-1-dependent manner. This study provides an integrated study including a cellular model, animal model, and tumor PDO, which provides evidence to understand the pathogenesis of CRC with BRAF-mutant, as well as new strategies for manipulation of CRC.

## Data Availability Statement

The raw data supporting the conclusions of this article will be made available by the authors, without undue reservation.

## Ethics Statement

The studies involving human participants were reviewed and approved by Research Ethics Committee of Renji Hospital. The patients/participants provided their written informed consent to participate in this study. The animal study was reviewed and approved by Research Ethics Committee of Renji Hospital.

## Author Contributions

MZ, HC, and JC conceived and designed the experiments. SS, SQ, and YL performed the experiments, analyzed the data, and wrote the manuscript. YH, RJ, and JS validated the manuscript. All authors read and approved the final manuscript.

## Conflict of Interest

The authors declare that the research was conducted in the absence of any commercial or financial relationships that could be construed as a potential conflict of interest.

## Supplementary Material

Supplementary Figure 1 BRAF inhibition restored AIM2 expression in CRC cell lines.
