# Supplementary figures and images for: AIM2 Inhibits BRAF-Mutant Colorectal Cancer Growth in a Caspase-1-Dependent Manner
Source: Front Cell Dev Biol. 2021 Mar 25;9:588278. doi: 10.3389/fcell.2021.588278 (PMC8027362; doi:10.3389/fcell.2021.588278)

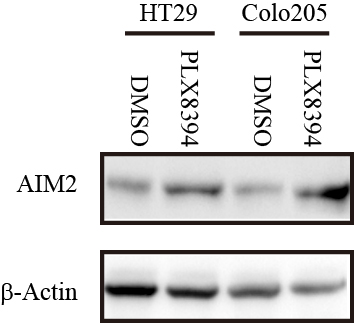

Supplement: Supplementary Figure 1 — BRAF inhibition restored AIM2 expression in CRC cell lines. [file Image_1.JPEG]
